# Post-transplant Diabetes Mellitus in Kidney Transplant Recipients in Sudan: A Comparison Between Tacrolimus and Cyclosporine-Based Immunosuppression

**DOI:** 10.7759/cureus.22285

**Published:** 2022-02-16

**Authors:** Elamein Yousif, Abdelrahman Abdelwahab

**Affiliations:** 1 Internal Medicine, Al Ahli Hospital, Doha, QAT; 2 Department of Nephrology, Ahmed Gasim Hospital, Khartoum, SDN

**Keywords:** nodat, sudan, cyclosporine, tacrolimus, kidney transplant, post-transplant diabetes mellitus

## Abstract

Hyperglycemia is a common complication among transplant patients without a history of diabetes mellitus (DM). Although new and potent immunosuppressants have improved short and long-term outcomes after transplantation, these drugs themselves may be associated with a greater risk of hyperglycemia. The present study aimed to determine the prevalence of post-transplant diabetes mellitus (PTDM) in post renal transplant patients in Sudan, and compare the effect of cyclosporine and tacrolimus-based immunosuppressive regimens. All adult kidney transplant recipients without pre-transplant diabetes who attended the transplant clinic at Ahmed Gasim Cardiac Surgery and Renal Transplant Center in Sudan were included. A total of 100 cases with functioning kidney allografts were enrolled in this study. The majority of cases were in the age range between 20 and 60 years (92%). Males and females were nearly equally distributed (56% vs 44%). Fifty-two percent of patients were on cyclosporine and 48% on tacrolimus. Overall, 18% of patients suffered from post-transplant diabetes mellitus. There was no statistically significant difference between tacrolimus and cyclosporine with regards to the prevalence of hyperglycemia (16.6% versus 13.4%; p>0.05).

## Introduction

It is well-known that diabetes mellitus (DM) is one of the most important causes of end-stage renal disease (ESRD) worldwide and renal transplantation is the treatment of choice for a significant proportion of patients with end-stage renal disease (ESRD) [1]. Post-transplant diabetes (PTDM), otherwise known as new-onset diabetes after transplant (NODAT), has been identified as a common and severe complication after renal transplantation [2]. In recent years, there has been an escalating interest in PTDM owing to its association with increased morbidity and the negative impact on patient and graft survival [3]. Interestingly, hyperglycemia is also a common complication among transplant patients without a history of DM, as the new potent immunosuppressant agents are known to cause hyperglycemia. Thus, in spite of their effect of improved short-term and long-term outcomes after transplantation, these drugs may adversely impact the patient’s survival by increasing blood sugar levels. PTDM, which is a combination of decreased insulin secretion and increased insulin resistance, is a strong, independent predictor of allograft failure and poor patient survival [4].

In a study done in 2007, among newly-listed adults for kidney transplantation, the overall prevalence of diabetes ranged from 40 to 65%. Additionally, about 4% to 25% of non-diabetic kidney transplant recipients were found to develop post-transplant diabetes mellitus (PTDM) per year [5]. In a recent study by Malik et al. (2021), PTDM was reported in 29% of post-kidney transplant patients [6]. The incidence of PTDM is variable (10 to 46%) depending on many factors such as study design and definition. Depending on the type of transplant, it occurs in 4-25% of renal transplant recipients, 2.5-25% of liver, 4-40% of heart, and 30-35% of lung transplant recipients [4]. Multiple risk factors have been implicated in the development of PTDM. It is worthwhile to note that PTDM has been reported to have a higher frequency among recipients receiving tacrolimus compared with cyclosporine [7].

Need for study

A number of large studies compared the cumulative incidence of PTDM in tacrolimus and cyclosporine-treated patients and showed that tacrolimus was associated with a higher relative risk of PTDM when compared to cyclosporine [8-10]. Studies have reported an incidence of 4.7% in patients receiving cyclosporine versus 11.5% of those on tacrolimus-based immunosuppression, post-renal transplant [10]. In contrast, a study from Romania found that PTDM was higher in a group of post-transplant patients on cyclosporine-based immunosuppression as opposed to those on tacrolimus [11]. Thus, there is a strong possibility that the race and ethnicity of the patients may play an important role in determining the response to immunosuppressive treatment. Current studies point toward switching over from tacrolimus to cyclosporine in those at high risk for PTDM [12]. However, it may be argued that non-white patients who are at higher risk of PTDM in view of their ethnicity are also the ones most likely to benefit from tacrolimus [13-14]. Hence it is prudent to establish the relationship between the two immunosuppressive drugs in this population.

Definition of PTDM [15]

According to International Consensus Guidelines, the diagnosis of PTDM can be made using any of the following criteria once the transplant recipient has been discharged from the hospital and tapered to their maintenance immunosuppression. However, glycated hemoglobin (HbA1c) should not be used alone to screen for a diagnosis of PTDM within the first year after transplant [12]. The criteria are: 1) Fasting glucose >126 mg/dL (7 mmol/L) on more than one occasion; 2) Random glucose >200 mg/dL (11.1 mmol/L) with symptoms; 3) Two-hour glucose after a 75-g OGTT of >200 mg/dL (11.1 mmol/L); 4) HbA1c >6.5%.

## Materials and methods

Aims and objectives

These include: To determine the prevalence of hyperglycemia among kidney transplant recipients at Ahmed Gasim center, Sudan; to analyze the effect of various demographic factors (age, gender, and educational level) on the development of hyperglycemia; and to compare the prevalence of hyperglycemia between the two major immunosuppressant drugs: cyclosporine and tacrolimus.

Study setting

The present study was a cross-sectional, hospital-based, descriptive study conducted in the transplant referral clinic at Ahmed Gasim Center, which is a governmental hospital in Khartoum North, Sudan, offering outpatient care, renal inpatient care, as well as equipped with an intensive care unit, with state-of-the-art diagnostic and therapeutic facilities. A total of 157 kidney transplant surgeries were carried out during the year 2016. The study was carried out after appropriate approval from the Institutional ethical committee.

Inclusion criteria

The study included all patients aged 18 years or older with a functioning kidney allograft, maintained on a low dose of prednisolone, and who attended Ahmed Gasim Transplant Clinic over a period of 12 months (January to December 2016).

Exclusion criteria

The study excluded all patients with a known history of pre-transplant diabetes and those who had a previous history of other organ transplantation.

Data collection/documentation

Data were collected using a self-administered structured pre-tested questionnaire, which included basic demographic details, year of transplant, symptoms of diabetes, immunosuppressive regimen, and duration of immunosuppression. Informed consent was obtained from all the participants, and confidentiality of data was assured. The study did not interfere with patient management.

Statistical analysis

Statistical analysis was done using SPSS version 20 (IBM Corp., Armonk, NY). Descriptive statistics were obtained for all study variables. All categorical variables were compared using the chi-square and Fisher’s exact tests and continuous variables were analyzed using the student’s t-test. All data were expressed as mean (±SD). A p-value < 0.05 was considered statistically significant.

## Results

The study included 100 patients who were recipients of kidney allograft and attended Ahmed Gasim Transplant Referral Clinic over a period of 12 months. It was aimed at determining the prevalence of hyperglycemia in post-transplant patients, to establish a comparison between the two major immunosuppressant regimens (cyclosporine and tacrolimus), as well as to determine the effect of the demographic factors on the development of hyperglycemia in these renal allograft patients.

Baseline characteristics of the study population

The general baseline characteristics of the patients are summarized in Table 1.

**Table 1:** Baseline characteristics

Demographic characteristic		Number (%)
Age(years)	18-40	46 (46)
	41-60	46 (46)
	>60	8 (8)
Gender	Male	56 (56)
	Female	44 (44)
Residence	Khartoum	17 (17)
	Khartoum North	15 (15)
	Omdurman	16 (16)
	Outside Khartoum	52 (52)
Education	Illiterate	26 (26)
	Basic education	41 (41)
	Higher education	33 (33)
Year of transplant	2012-2017	54 (54)
	2005-2011	42 (42)
	1998-2004	4 (4)
Immunosuppressive regimen	Cyclosporine	52 (52)
	Tacrolimus	48 (48)
Duration of immunosuppression (years)	1-6	70 (70)
	7-12	20 (20)
	13-18	4 (4)
	>18	2 (2)
Symptoms of diabetes	Increased urination	15 (15)
	Increased thirst	14 (14)
	Increased hunger	9 (9)
	All of the above	31 (31)
PTDM	Yes	18 (18)
	No	82 (82)

The majority of the cases were in the age range of 18-60 years (92%). Fifty-six percent (56%) of the cases were males and most of the patients were from outside Khartoum (52%). With reference to education, 41% had a basic education level while 33% had a higher education level and only 26% were illiterate. A majority of the participants were recently transplanted between the years 2012 and 2017 (54%) while 42% were between the years 2005 and 2011 and 4% received their transplant between the years 1998 and 2004. Fifty-two percent (52%) of the patients were using cyclosporine and 48% were using tacrolimus-based immunosuppressive regimens. Seventy percent of the cases had been on immunosuppressive therapy for one to six years.

Laboratory parameters

The patients were tested for their fasting blood sugar (FBS) and HbA1c. The results of these are summarized in Table 2.

**Table 2:** Prevalence of hyperglycemia HbA1c: glycated hemoglobin

Variable		Number (%)
Fasting blood sugar (mg/dL)	70 – 126	82 (82)
	>= 127	18 (18)
HbA1c (%)	<= 6.5	69 (69)
	> 6.5	31 (31)

Eighty-two percent (82%) had normal FBS between 70 and 126 mg/dl and 18% were between 127 and 146mg/dl. HbA1c levels were less than 6.5% in 69% of cases and elevated (>6.5%) in 31%.

Comparison between baseline parameters and glycemic status

The results of the comparison between the baseline variables, such as age and gender, are shown in Table 3. One of the main aims of the study was to determine the difference in the prevalence of hyperglycemia between those on cyclosporine and tacrolimus, the results of which are also presented in the table.

**Table 3:** Comparison of hyperglycemia with baseline variables and immunosuppressive treatment

Variable		Fasting blood sugar (mg/dl)		P-value
		70 - 126	>= 127	
Age (years)	18-40	41	5	0.49
	41-60	37	9	
	>60	7	1	
Gender	Male	46	10	0.53
	Female	39	5	
Drug	Cyclosporine	45	7	0.86
	Tacrolimus	40	8	

There was no statistically significant relationship between the fasting blood sugar and the age or gender of the patients.

It was also noted that although there was a small increase in the prevalence of hyperglycemia among patients on tacrolimus as compared to cyclosporine (16.6% vs 13.4%), this was not statistically significant.

## Discussion

This study confirms that hyperglycemia (PTDM) is highly prevalent (18%) in Sudanese post-renal transplant patients.

The results of the present study concur with a retrospective study of 221 Sudanese patients who received live donor kidney transplantation in Ahmed Gasim Kidney Transplant Center between December 2001 and December 2007, in which the reported 12-month cumulative incidence of PTDM in the transplant population was 17.6% [9]. Similarly, in a study in Japan, where 127 post-transplant patients were followed up, the incidence rate of PTDM was 15.1% in Japanese kidney transplant recipients [16].

On the contrary, a systematic review of the literature was done in 2004 to determine the incidence of PTDM, which included 19 studies with 3,611 patients. The 12-month cumulative incidence of PTD was much lower (<10% in most studies) [17]. One reason for this wide variation in reported prevalence may be due to the challenges faced in making the diagnosis.

We studied the demographical data of each participant, and the result revealed that the majority of the cases were ranging between the ages of 18 and 60 (92%). However, there was no statistically significant relationship between age and the development of hyperglycemia. A review of the literature regarding the impact of age on PTDM shows conflicting results. While certain studies found that age was an independent risk factor for PTDM, some others reported no impact. Numakura et al. reported that in Japanese patients, age over 50 was associated with the development of PTDM [18]. Similarly, a French study also demonstrated age to be a strong risk factor [19]. In contrast, Yu et al., in a study of Korean patients, found an odds ratio of just 1.05 in older patients (95 % confidence interval [CI]: 1.01-1.08) [20]. The variability in different studies on the impact of age may be due to the fact that each of these studies has been carried out in different ethnic groups. From the present study, it may be observed that age has no significant impact on the development of PTDM in Sudanese patients.

Males were more than females (56% v 44%), however, there was no significant relationship between gender and post-transplant hyperglycemia. A similar lack of association was found in most studies [21].

In this study, with regard to the date of the renal transplant, the results show that the majority of transplants were recent, between the years 2012 and 2017 (54%), 42% were between the years 2005 and 2011, and only 4% were between 1998 and 2004. The study did not find any relationship between the time since transplantation and hyperglycemia. One long-term study conducted between 1976 and 2004 in Egypt demonstrated a diagnosis of PTDM in 18.2% of patients overall, in whom 52.4% were diagnosed by six months and 11.5% between six and 12 months [22]. A French study by Kamar et al. indicated a median time-to-onset of 1.6 months, with an incidence of 7% over 2 years [19]. In the United States, using data from the United Renal Data System, the cumulative incidence of PTDM was 9.1%, 16.0%, and 24% at three, 12, and 36 months post-transplant, respectively [5].

With regard to the blood sugars of the patients in this study, the fasting blood sugar levels were normal (between 70 and 126 mg/dl) in 82% of patients, and high in 18%. The HbA1c level was found to be normal in 69% and was high in 31%, but HbA1c had not been routinely employed at most transplant centers.

One of the primary objectives of the current study was to evaluate the differences between the patient groups on cyclosporine and tacrolimus-based immunosuppressive therapy. It was found that 52% and 48% of the patients were on cyclosporine and tacrolimus, respectively. According to the duration for which the patients had been using the drug, 70% were using it for 1-6 years, 20% between 7-12 years, 4% for 13-18 years, and only 2% for 19 years or more.

On comparing tacrolimus to cyclosporine, the percentage of patients developing hyperglycemia was slightly higher by 3% in those receiving tacrolimus, however, there was no statistically significant difference. This is unlike the previous studies where tacrolimus is considered to be more diabetogenic by about 50% [23]. Initial studies comparing cyclosporine (CsA) and tacrolimus in kidney transplant recipients demonstrated higher PTDM risk with tacrolimus therapy [24-26]. The comparatively greater diabetogenicity of tacrolimus was also supported by analysis of USRDS data, which specified that tacrolimus was associated with a 48% to 66% increase in PTDM risk by two years after transplantation compared with CsA [27]. However, subsequent studies of combination immunosuppression with tacrolimus and mycophenolate mofetil documented reduced rates of hyperglycemia when compared with the initial reports, most likely attributable to tacrolimus-sparing effects with more potent adjunctive therapy [28]. On the other hand, a study from Romania found that PTDM was higher in a group of post-transplant patients on cyclosporine-based immunosuppression as opposed to those on tacrolimus [11]. These results are at the opposite end of the spectrum. In our study of Sudanese patients, there was no statistically significant association found between the groups of CsA and tacrolimus in the prevalence of PTDM. Thus, there is a strong possibility that the race and ethnicity of the patients may play an important role in determining the response to immunosuppressive treatment.

## Conclusions

The prevalence of post-transplant diabetes mellitus (PTDM) in adult kidney transplant recipients at Ahmed Gasim Referral Clinic in Sudan was found to be 18%. Of these, 48% were on tacrolimus-based immunosuppression and 52% on cyclosporine. The difference in hyperglycemia was not statistically significant between the two groups (16.6% in the tacrolimus group vs. 13.4% in the cyclosporine group). Further, demographic factors, such as age and gender, were not found to have any significant association with the development of hyperglycemia in the study population. The findings of the study may represent the fact that the hyperglycemic response of the patients to immunosuppression is likely to be different in the population with non-white ethnicity.
